# A preliminary prediction model for potentially guiding patient choices between breast conserving surgery and mastectomy in early breast cancer patients; a Dutch experience

**DOI:** 10.1007/s11136-017-1740-0

**Published:** 2017-11-17

**Authors:** Elvira Vos, Linetta Koppert, Winnifred van Lankeren, Cornelis Verhoef, Bas Groot Koerkamp, Myriam Hunink

**Affiliations:** 1 Department of Surgery, Erasmus MC Cancer Institute, Rotterdam, The Netherlands; 2 Department of Radiology, Erasmus MC, Rotterdam, The Netherlands; 3 Department of Epidemiology and Department of Radiology, Erasmus MC, Rotterdam, The Netherlands; 4 Center for Health Decision Science, Harvard T.H. Chan School of Public Health, Boston, USA; 5 Department of Oncological Surgery, Erasmus MC Cancer Institute, DHA-102, PO Box 5201, 3008 AE Rotterdam, The Netherlands

**Keywords:** Treatment decision making, Breast cancer surgery, Breast conserving surgery, Mastectomy, Breast reconstruction, Cosmetic result

## Abstract

**Purpose:**

To guide early stage breast cancer patients to choose between breast conserving surgery (BCS) and mastectomy (MST) considering the predicted cosmetic result and quality of life (QoL).

**Methods:**

A decision model was built to compare QoL after BCS and MST. Treatment could result in BCS with good cosmesis, BCS with poor cosmesis, MST only, and MST with breast reconstruction. QoL for these treatment outcomes were obtained from a previous study and the literature and translated into EuroQoL-5D derived utilities. Chance of good cosmesis after BCS was predicted based on tumor location and tumor/breast volume ratio. The decision model determined whether the expected QoL was superior after BCS or MST based on chance of good cosmesis.

**Results:**

The mean utility for the treatments such as BCS with good cosmesis, BCS with poor cosmesis, MST only, and MST with breast reconstruction were 0.908, 0.843, 0.859, and 0.876, respectively. BCS resulted in superior QoL compared to MST in patients with a chance of good cosmesis above 36%. This 36% threshold is reached in case the tumor is located in the upper lateral, lower lateral, upper medial, lower medial, and central quadrant of the breast with a tumor/breast volume ratio below 21.6, 4.1, 15.1, 3.2, and 14.7, respectively.

**Conclusions:**

BCS results in superior QoL in patients with tumors in the upper breast quadrants or centrally and a tumor/breast volume ratio below 15. MST results in superior QoL in patients with tumors in the lower breast quadrants and a tumor/breast volume ratio above 4.

**Electronic supplementary material:**

The online version of this article (10.1007/s11136-017-1740-0) contains supplementary material, which is available to authorized users.

## Introduction

Early stage breast cancer patients and their surgeons are confronted with the complex decision between breast conserving surgery (BCS) with radiotherapy or mastectomy (MST) with or without breast reconstruction. Both have similar overall survival [1–3]. In the absence of an oncological contraindication for BCS, the treatment choice is a matter of expected cosmetic result that influences quality of life (QoL) [4, 5]. The surgeon and patient discuss the expected cosmetic result of both BCS and MST, but preoperative prediction of the cosmetic result is typically based on informal assessment by the surgeon. An objective decision aid taking cosmesis and QoL into consideration does not exist.

It is generally believed that the benefit of BCS over MST depends on the cosmetic result after BCS. Good cosmesis after BCS has shown to yield a substantial QoL benefit over poor cosmesis [4]. Therefore, it can be hypothesized that BCS is preferable when the chance of a good cosmetic result is high, but MST (with or without breast reconstruction) is preferable when BCS is unlikely to have a good cosmetic result. We have previously demonstrated that a good cosmetic result after BCS can be predicted preoperatively by tumor volume/breast volume ratio (TV/BV ratio) and tumor location [6]. It remains unknown above what specific chance of good cosmetic result, BCS results in higher QoL over MST. If BCS is already performed for a low chance of good cosmesis, too many patients receive BCS increasing the incidence of poor cosmesis and reducing QoL. If BCS is only performed in those patients where good cosmesis is very likely and MST is performed otherwise, too many patients receive MST reducing QoL.

The aim was to guide decision making for patients with early stage breast cancer using a decision model that considers both predicted cosmetic result and QoL after BCS and MST. The treatment threshold—when to treat with BCS or MST—for optimal QoL is calculated. In clinical practice, this decision model could inform the treatment decision by weighing QoL of each treatment option.

## Methods

### Study population

Data from a study population of 69 patients previously described in more detail formed the basis for the current analysis [6]. During first year, all consecutive patients who came for follow-up visit at the outpatient clinic at the Erasmus MC Cancer Institute were asked to participate. Eligibility criteria included: Females above 18 years old with BCS and adjuvant radiotherapy between 2007 and 2012 for an invasive breast cancer with a preoperative MRI available. Preoperative MRI was an inclusion criterion to allow accurate breast volume measurements. BCS was performed using basic oncoplastic techniques, but no volume replacement techniques or therapeutic reduction mammoplasty. Patients with MST were not eligible since at first we only aimed to build a prediction model for cosmetic result after BCS. Informed consent was obtained from all participants.

The study population from Jagsi et al. consisted of woman aged between 20 and 79 years with stage 0–III breast cancer diagnosed between 2005 and 2007 and treated with MST only (*n* = 263) or MST with reconstruction (both implant and autologous, both immediate and delayed) (*n* = 222) from the Los Angeles and Detroit population based Surveillance, Epidemiology, and End Results registries. These were the patients that responded to a survey including QoL questionnaire at 9 months after diagnosis and approximately 4 years later constituting a 73 and 68% response rate [7]. Patient characteristics of both study populations are shown in Table 1.

**Table 1:** Patient and treatment characteristics of the two study populations from whom the QoL values were translated into EQ-5D derived utilities

	BCS^a^ (*n* = 69)	Mastectomy^b^ (*n* = 485)	*P* value
Age (years)			0.072
≤ 45	8 (11.6)	99 (20.4)	
46–55	28 (40.6)	140 (28.9)	
≥ 56	33 (47.8)	246 (50.7)	
Stage			< 0.001
0	–	99 (20.4)	
I	45 (65.2)	133 (27.4)	
II	20 (29.0)	164 (33.8)	
III	1 (1.0)	87 (17.9)	
Missing	3 (4.3)	2 (0.4)	
Radiotherapy			< 0.001
Yes	69 (100)	144 (29.7)	
No	–	322 (66.4)	
Missing	–	19 (3.9)	
Chemotherapy			0.054
Yes	31 (44.9)	278 (57.3)	
No	38 (55.1)	198 (40.8)	
Missing	–	9 (1.9)	

^a^Our study population for the health states BCS with good cosmetic result and BCS with poor cosmetic result

^b^Study population from Jagsi et al. [7] for the health states Mastectomy only and Mastectomy with breast reconstruction

### Tumor volume/breast volume ratio and tumor location

The preoperative MRI datasets were transferred to a four-walled CAVE™-like virtual reality system (i.e., the I-Space), at the department of Bioinformatics, Erasmus MC. The volume rendering application creates an interactive ‘hologram’ of the MRI and reliably performs volume measurements based on greyscale differences [8]. Interclass and intraclass correlation coefficients for tumor and breast volumes all exceeded 0.95 [6]. Tumor volume/breast volume ratio (TV/BV ratio) was calculated by dividing the tumor volume in cm^3^ by the breast volume in cm^3^ and multiplied by 1000. Details about the TV/BV ratio were published elsewhere [6]. The tumor location was determined by the radiologist and designated to one of the four quadrants of the breast or a central position.

### Cosmetic result evaluation

Cosmetic result was determined by panel assessment of post-radiotherapy photographs. The panel consisted of a plastic surgeon, general surgeon, radiotherapist, general practitioner, layperson, and breast cancer patient. They each scored the four photographs taken by a professional medical photographer in a standardized manner. The self-developed ‘Erasmus MC Panel questionnaire’ (Online Resource 1) consisting of eleven items was scored on a four-point scale (0 = excellent, 1 = good, 2 = moderate, 3 = poor) [6]. If the average score of all items and panel members was below or equal to 1.5, it was categorized as a good cosmetic result. In case the average score was above 1.5, it was categorized as a poor cosmetic result.

### Cosmetic result prediction model

In the study population of 69 BCS patients, a preoperative prediction model for good cosmetic result after BCS was built [6]. It included the two independent and statistically significant predictors, namely TV/BV ratio (on a continuous scale) and tumor location (categorical variable with upper lateral (reference), upper medial, lower lateral, lower medial quadrant, and central). The following parameters were not independently associated with cosmetic result and therefore not included in the prediction model: age, tumor diameter, excision volume, presence of adjacent DCIS, use of re-excision, and time of follow-up. Specimen weight was excluded from the analyses, because it was not preoperatively available. The analysis for cosmetic result predictors can be found in Online Resource 2.

**Fig. 1:**
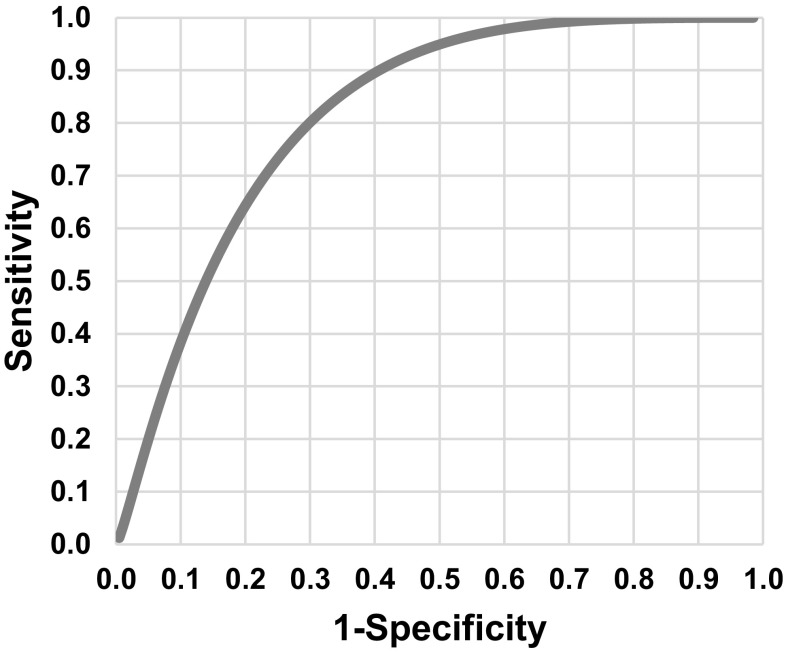
Smooth ROC curve and equation of the prediction model for the chance of a good cosmetic result after breast conserving surgery (BCS). The prediction model combines the individual patient's tumor volume/breast volume ratio and location of the tumor in the breast. Area under the operating characteristic curve (AUC) was 0.83 (95% CI 0.71–0.94)

The prediction model result (R) is the chance of a good cosmetic result after BCS. The original formula of R is shown in Online Resource 3, equation 1. The prediction model inherently has true positives (i.e., good cosmesis is predicted which is true), false positives (i.e., good cosmesis is predicted but not true), true negatives (i.e., poor cosmesis is predicted which is true) and false negatives (i.e., poor cosmesis is predicted but not true) (Fig. 1). However, a *threshold* at which BCS is preferred— at what chance of good cosmesis it is better to perform BCS rather than MST—is unknown. The threshold at which BCS is preferred can be established by modeling the harms and benefits in QoL for each treatment approach.

### Treatment decision tree

**Fig. 2:**
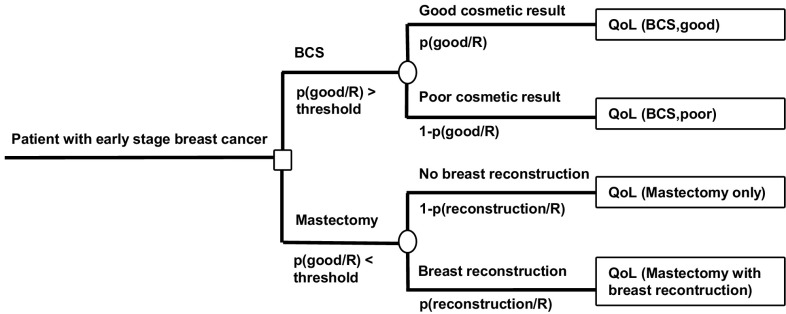
Treatment decision tree in early staged breast cancer patients comparing BCS and MST. The model determines when BCS is superior to MST depending on the chance of a good cosmetic result and the postoperative QoL. The chance of a good cosmetic result is based on the individual patient's tumor volume/breast volume ratio and tumor location which is calculated by our prediction model (R). The treatment threshold for the chance of a good cosmetic result is reached when the QoL of BCS and MST are equal; BCS is preferred for values above the threshold and MST is preferred for values below the threshold. *BCS* breast conserving surgery, *p* probability, *R* result of prediction model for cosmetic result after BCS, *QoL* quality of life

A treatment decision tree was developed to model the treatment consequences of the prediction model and the prediction model inherent uncertainty (Fig. 2). Patients entered the tree having the diagnosis of invasive breast cancer stage I–IIIA opting for BCS. Each endpoint in the decision tree represents a ‘health state’, namely the health states BCS with good cosmesis (i.e., true positive result), BCS with poor cosmesis (i.e., false positive result), mastectomy only, and mastectomy with breast reconstruction (i.e., both a negative result). Note that after a MST, it is impossible to distinguish between a true negative result (i.e., poor cosmesis if BCS had been performed) and a false negative result (i.e., good cosmesis if BCS had been performed). We assumed good cosmesis after BCS is preferred over MST (± reconstruction) and MST (± reconstruction) is preferred over poor cosmesis after BCS. It was also assumed that QoL after BCS is related to the cosmetic result. The probability of the health state BCS with good cosmesis equaled the prediction model probability for good cosmesis. The opposite from this equaled the probability for BCS with poor cosmesis. The probability of breast reconstruction was estimated from the most recent study with breast reconstruction rate as their primary outcome [9]. The opposite of this equaled the probability for MST only.

### Quality of life

The primary outcome of each health state in the decision tree was a long-term QoL weight from the patient’s perspective. Since life expectancy is equivalent for MST and BCS [1–3], the primary outcome measure QoL was used instead of the quality-adjusted life-years. QoL was defined as a utility, which is a single index-based value between 0 and 1 that enables assessing the effects of interventions on health-related quality of life. In contrast to most patient-reported outcome measures, the EuroQoL 5D (EQ-5D) offers utilities [10]. Making use of time trade-off experiments, preference weights (tariffs) have been determined, and each health state of the EQ-5D can be assigned an index score. Freedman et al. describes the largest population (*n* = 1050) with EQ-5D derived utilities at 5 years after BCS in the literature [11]. However, no distinction was made in cosmetic result. To obtain a robust utility for good and poor cosmesis, from our study population the gain and loss in utility for a good and poor cosmesis was added to the mean utility after BCS from Freedman et al. (analysis are shown in Online Resource 4). The EORTC QLQ-C30 values from our study population were converted into EQ-5D compatible utilities making use of the ‘map’ from Crott et al. (ordinary least squares regression model 3) [12, 13]. For the health states involving MST, the most recent and representative QoL values from the literature by Jagsi et al. were used [7]. The FACT-G QoL values from the study population of Jagsi et al. were converted into EQ-5D compatible utilities making use of the map from Teckle et al. (ordinary least squares regression model 2) [14]. All raw QoL values and equations for conversion to EQ-5D utilities can be found in Online Resource 4.

### Treatment threshold

The definition of a treatment (treat—do not treat) threshold is the probability of disease at which the expected value of treatment and no treatment are equal [15]. When applied to the current study, the treatment threshold—to perform BCS or not—was defined by the chance of good cosmetic result after BCS at which the QoL after BCS and the QoL after MST (± breast reconstruction) was equal. The threshold can be determined by direct comparison of the benefits and harms of BCS. The benefit is defined as the gain in QoL from living with a good cosmetic result after BCS instead of a MST (± breast reconstruction) (Online Resource 3, equation 2A). The harm is defined by the loss in QoL from living with a poor cosmetic result after BCS instead of a MST (± breast reconstruction) (Online Resource 3, equation 2B).

BCS should be performed when the chance of good cosmesis after BCS exceeds the harm to benefit ratio as presented in the Online Resource 3, equation 3A. Rewriting this expression as probability and substituting equation 2A, B leads to equation 3B, C [16]. The treatment threshold was determined by inserting the utilities in the equation.

### Data analysis

The prediction model for good cosmetic result after BCS developed in our study population consists of (1) regression coefficients representing the influence of the variables TV/BV ratio and tumor location on the (log odds of the) probability of good cosmesis and (2) an intercept representing the prior probability of good cosmesis in the study population. No random effects were included in the prediction model. The models predictive ability was estimated by area under the operating characteristic curve (AUC). However, the prior probability was assumed to be higher than the actual prevalence of good cosmesis since women with a false negative prediction model outcome (i.e., poor cosmesis is predicted but not true) underwent MST and therefore, were not included in the study population; even though a good cosmetic result was expected if BCS had been performed. The number of false negatives is unknown. Therefore, a prior probability slightly larger (2%) than the actual prevalence in the study population was chosen to correct for these false negatives and the intercept of the updated prediction model was recalculated (Online Resource 3, equation 4 and Fig. 1).

A probabilistic sensitivity analysis (PSA) was performed to evaluate the joint effect of uncertainty about all parameters in the model [17]. Parameter uncertainty was expressed by beta probability distributions on the interval [0, 1]. Thereafter, 10,000 times random values from the distributions of all variables were drawn and the model was recalculated for each set (sample) of values using an average patient, resulting in a probability distribution of the expected outcome (i.e., average expected utility for BCS and MST). The average patient was determined by calculating the study population mean for each parameter included in the prediction model. Subsequently, the expected benefit in utility per patient in the absence of parameter uncertainty (i.e., expected value of perfect information, EVPI) was determined. In each sample, the opportunity loss was defined as the difference in utility between the maximum expected benefit of that sample (BCS or MST) and the sample’s expected benefit of the baseline optimal treatment (BCS) [17]. *P* values were derived from two-tailed tests and *P* < 0.05 was considered significant. Microsoft Excel 2010 and IBM SPSS Statistics version 22 were used for all analyses. There was no funding support for this study.

## Results

### Tumor volume/breast volume ratio and tumor location

In the 69 breast cancer patients with preoperative MRI and treated by BCS, the median TV/BV ratio was 2.47 (IQR 1.25–5.54). Thirty-three (47.8%) tumors were located in the upper lateral quadrant, 14 (20.3%) tumors in the upper medial quadrant, 13 (18.8%) tumors in the lower lateral quadrant, 3 (4.3%) tumors in the lower medial quadrant, and 6 (8.7%) tumors in a central position.

### Chance of good cosmetic result

Median time between surgery and cosmetic result assessment was 33 months (interquartile range 18–48). The panel evaluation of cosmetic result resulted in 49/69 (71.0%) patients with good cosmesis (i.e., Erasmus MC Panel score ≤ 1.5) and 20/69 (29.0%) patients with poor cosmesis (i.e. , Erasmus MC Panel score > 1.5). The median chance of good cosmetic result from the prediction model was 0.84 (interquartile range 0.45–0.92). The formula of the prediction model is depicted in Fig. 1. The prediction model had an area under the operating characteristic curve (AUC) of 0.827 (95% CI 0.71–0.94) meaning that in 82.7% of the cases, it correctly discriminated between poor and good cosmesis (Fig. 1). Other patient and treatment characteristics are shown in Table 1. Four oncological surgeons almost exclusively performing breast surgery operated 61/69 (88.4%) of the patients and 8/69 (11.6%) of the patients were operated by general oncological surgeons.

### Quality of life

Of the 69 patients with BCS included, 61 (88.4%) responded to the EORTC QLQ-C30 questionnaire to evaluate their QoL at a median of 38 months (interquartile range 31–54) after surgery. From the respondents, 44 had good cosmetic result and 17 had poor cosmetic result. Utilities are presented in Table 2 and their calculations are shown in Online Resource 4. The mean utility for poor cosmesis and good cosmesis were not statistically significantly different (*P* = 0.055). After linear regression analysis, QoL was not statistically significantly associated with increasing age (coefficient 0.002 95% CI − 0.002 to 0.006), higher breast cancer stage (coefficient − 0.008 95% CI − 0.070 to 0.054), and adjuvant chemotherapy (coefficient − 0.018 95% CI − 0.080 to 0.044).

**Table 2:** Utilities

BCS, good cosmetic result	0.908
BCS, poor cosmetic result	0.843
Mastectomy only	0.859
Mastectomy with breast reconstruction	0.876
Mastectomy (± reconstruction)	0.866

Jagsi et al. included 263 patients with MST only and 222 patients with MST plus reconstruction (see patient characteristics Table 1) who completed the FACT-G that was measured at a mean of 50 months (standard deviation 5) after diagnosis (see utilities in Table 2 and calculations in Online Resource 4).

### When to treat with BCS or MST for superior QoL

**Fig. 3:**
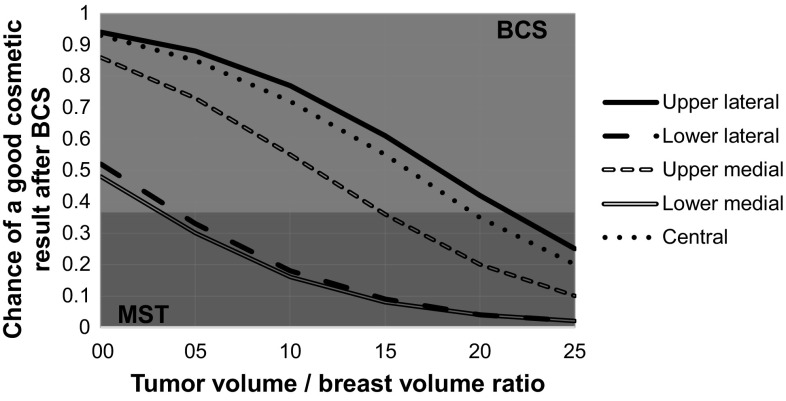
Decision graph to determine whether the expected QoL after surgery is better with BCS (green zone) or MST (red zone). The decision is based on the chance of a good cosmetic result. The decision model demonstrated that QoL after BCS is superior if the chance of a good cosmetic result exceeds 36%. The chance of a good cosmetic result varies between patients and depends on the location of the tumor and the ratio of tumor volume and breast volume. For example, a patient with a lower lateral tumor and a volume ratio of 15 has a low chance of a good cosmetic result after BCS (about 10%) and should therefore consider undergoing MST. On the other hand, a patient with an upper lateral tumor and the same ratio of 15 has a 60% chance of a good cosmetic result after BCS (about 60%) and should consider BCS. *BCS* breast conserving surgery, *MST* mastectomy (with or without breast reconstruction). (Color figure online)

The benefit in utility from performing BCS with a good cosmetic result rather than a MST (± reconstruction) was: 0.908 − 0.866 = 0.042 (Table 2) (Online Resource 3, equation 2A). The harm in utility from performing BCS with a poor cosmetic result rather than a MST (± reconstruction) was: 0.866 − 0.843 = 0.023 (Table 2) (Online Resource 3, equation 2B). BCS should be performed if the chance of a good cosmetic result exceeds 35.8% (Online Resource 3, equation 6) (Fig. 3). For application in clinical practice, the following decision rule can be used. BCS is preferred for an upper lateral, lower lateral, upper medial, lower medial, and central tumor location in case the TV/BV ratio is below 21.6, 4.1, 15.1, 3.2, and 14.7, respectively (Fig. 3). From all 9 patients with a chance of good cosmesis below 35.8% (i.e., negative result) 8/9 had a TV/BV ratio above 5.0 and 7/9 had their tumor located in the lower lateral quadrant.

**Fig. 4:**
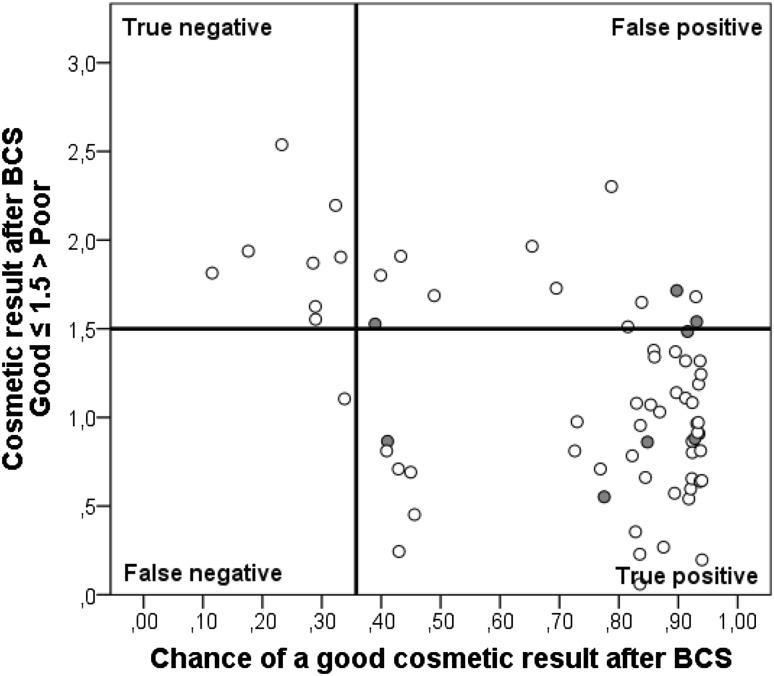
The predicted cosmetic results of the study population on the x-axis are plotted against the cosmetic result as evaluated by the panel on the y-axis. The horizontal line is set at a panel evaluation of cosmetic result cut-off score of 1.5. Values ≤ 1.5 are defined as good cosmesis and values > 1.5 as poor cosmesis. The vertical line is set at the treatment threshold of 0.36 resulting from the decision model. If the predicted cosmetic result is below the threshold, mastectomy (with or without reconstruction) results in the optimal QoL. If the predicted cosmetic result is above the threshold, BCS results in the optimal QoL. This figure shows the number of true positives (good cosmesis after BCS as predicted preoperatively), true negatives (poor cosmesis after BCS as predicted preoperatively), false positives (poor cosmesis after BCS but preoperative prediction was good), and false negatives (good cosmesis after BCS but preoperative prediction was poor) when the prediction model is applied to the study population. The eight patients operated by an—non breast cancer specific—oncological surgeon were colored in red. (Color figure online)

Figure 4 shows the number of true positives, true negatives, false positives and false negatives when the prediction model is applied to our study population. Nine out of 69 patients (13.0%) had a chance of good cosmesis below 35.8% (i.e., negative) and would have been advised against BCS had the results of our analysis been available. Of these 9 patients, 8 indeed had a poor cosmetic result (i.e., true negative) and 1 patient was classified as good cosmetic result (i.e., false negative). The corresponding negative predictive value of the prediction model was 88.9%. The false negative patient had a chance of good cosmesis of 33.8%, which was very close to the threshold. Of the 60 patients with a chance of good cosmesis exceeding 35.8% (i.e., positive), 48 patients indeed had a good cosmetic result (i.e., true positive) and 12 patients were classified as poor cosmetic result (i.e., false positive). The corresponding positive predictive value of the prediction model was 80.0%. The false positive patients had a chance of good cosmesis ranging between 39–93%.

### Parameter uncertainty

The average patient from our study population had a chance of good cosmetic result after BCS of 71%. For example, a patient with a tumor of 3.8 cm^3^ in the upper medial quadrant in a breast of 675 cm^3^ resulted in a TV/BV ratio of 5.6. Taken into consideration, the parameter uncertainty of our decision model, for this patient on average BCS was the treatment with the largest benefit in QoL and was advised in 61.9% of the 10,000 samples (see probabilistic sensitivity analysis in Online Resource 5). If we reduced our parameter uncertainty, the benefit in QoL would be 0.02 per patient (see expected value of perfect information analysis in Online Resource 5).

## Discussion

BCS is the treatment with superior QoL for breast cancer patients with a chance on good cosmetic result exceeding 36%. This 36% threshold is reached in case of an upper lateral, lower lateral, upper medial, lower medial, and central tumor location in combination with a tumor/breast volume ratio below 21.6, 4.1, 15.1, 3.2, and 14.7, respectively. In case of larger tumor/breast volume ratio’s, MST (with or without breast reconstruction) will result in higher QoL.

Due to the progress in surgical techniques and improving oncological outcomes, QoL and cosmesis have an increasingly prominent role in breast cancer treatment. Incorporating QoL and cosmesis in a treatment decision model comes with challenges due to their subjective entity. Cosmesis cannot be captured in clear-cut classifications and a (gold) standard for measuring cosmesis after BCS is lacking. Consequently, the validity of this decision model depends on the methodology of measuring and predicting cosmetic result and the definition of good and poor cosmesis. This is a weakness of our study since the validity of our definitions and self-developed Erasmus MC Panel questionnaire have not been tested extensively. The panel assessment is therefore study specific. How to measure cosmetic result after BCS is still a matter of debate, however, a multiple-person panel evaluation remains the most used and recommended [18]. It does come with disadvantages as it remains a subjective method with low reproducibility results influenced by the type of observers (experts versus non-experts) and the scale used. Consensus increases if the outcome is dichotomized. It could be argued that cosmetic result should be based on a patient-reported outcome measure (PROM). However, PROM’s only deliver scores on a continuous scale and their predictive ability is low (e.g., TV/BV ratio was not associated with patient evaluation of cosmetic result) [6]. The more efficient BCCT.core software for cosmetic result evaluation might become the future standard, but until today, agreement with panel evaluations differs [19, 20]. The 12 patients with false positive result (i.e., model predicted a good cosmetic result but the panel evaluated cosmesis as poor) could be explained by the fact that 8 out of 12 patients had visible radiotherapy fibrosis or skin color changes and retractions around the scar which is not taken into consideration in the predicted cosmetic result. Another explanation could be that 3/12 (25%) of false positive patients were operated by a general oncological surgeon (i.e., not breast cancer specific surgeon) as compared to 8/69 (12%) of the total study population. This suggests that surgeon experience matters for achieving a good cosmetic result. The lack of other significant parameters, like radiotherapy boost, in the prediction model for cosmetic result was a disadvantage. Yet the model proved to discriminate well between poor and good cosmetic result (AUC of 0.83). As far as we know, other prediction models for cosmetic result or other decision models including cosmetic result and/or QoL are not available in the literature. Therefore, our models could not be compared or tested for external validity.

QoL is a complex and multifactorial outcome that is not captured by cosmesis and type of surgery only. For example, age, chemotherapy, and fear of recurrence have been shown to be associated with QoL [21–23]. However, fear of recurrence was not considered here and no significant association was seen between QoL and age, stage, or chemotherapy. The lack of adjusted utilities for these factors was a drawback. Future studies should also address fear of recurrence. The mean utility found for poor and good cosmesis after BCS were not significantly different (*P* = 0.055). No sample size calculation was performed for this study and it was not powered to show a significant QoL difference. The nature of our tertiary referral university hospital caused a small study population for BCS. This had multiple implications, like the lack of power to detect QoL differences and large (statistical) uncertainty of the utilities for BCS. To decrease this uncertainty, the mean utility for BCS in general measured by EQ-5D in 1050 patients from Freedman et al. was used [11]. The results are, however, not directly generalizable beyond our study population and need to be validated. Due to the small sample size, the results of this study are preliminary. Work to validate the results in a larger study is underway.

It was assumed that QoL after BCS would be related to the cosmetic result. However, cosmesis was not significantly associated with EORTC QLQ C-30 and BR-23 scores, although absolute differences were seen [e.g., body image score of 87 vs. 73 (0–100) in good versus poor cosmesis, *P* = 0.203] [6]. This could be explained by the fact that all the questionnaires are general or disease-specific, not cosmetic result specific. The assessment of cosmesis after BCS by an independent panel, and the lack of a panel assessment after MST (with and without breast reconstruction), could also explain the lack of association with PROM’s. Studies with patients’ cosmetic evaluation, however, were significantly associated with QoL [4, 5]. A larger study population is needed to draw final conclusions about whether EQ-5D is sensitive for breast cancer surgery outcome differences. Promising alternatives for future efficiency analysis are the recently published EORTC Quality of Life Utility Measure-Core 10 dimensions (QLU-C10D) [24], EORTC Quality of Life for women undergoing breast reconstruction (QLQ-BRECON26) [25], and the modernized EORTC BR23 module that is currently under development.

Another disadvantage was the complexity and use of different sources for generating the utilities. Namely, EORTC values with EQ-5D UK tariffs from our study population, EQ-5D values with unknown origin of tariffs from Freedman et al. and FACT values with EQ-5D US tariffs from Jagsi at el. The time of measurement was 38 months after surgery, 60 months after surgery, and 50 months after diagnosis, respectively. We did not correct for these time differences. Neither did we correct for the variety in study population size, but the standard deviations in the PSA show that the uncertainty for most BCS related parameters is larger when compared to the MST-related parameters caused by a smaller study population. The mastectomy group had more stage III patients (1 vs. 18%) who underwent less radiotherapy (100 vs. 30%), but this was not unexpected. An alternative for using multiple sources to define the utilities for each health state was not available since, as far as we know, no study has been published presenting EQ-5D derived QoL values for the different surgical treatments of breast cancer. We were the first to compare utilities between BCS and MST.

Whether the differences in utilities found between the treatment options are clinically meaningful can be questioned. The minimally important difference has been estimated to be 0.08 for UK-based EQ-5D scores and 0.06 for US-based EQ-5D scores by Pickard et al [26]. We found a difference between good and poor cosmetic result after BCS of 0.065. However, Pickard et al. estimated the minimally important differences in advanced stage patients with cancers from all kinds of primary origin. Our study population consists of early stage breast cancer patients that already have a high baseline QoL (towards the maximum of one) which may impede finding large QoL differences. Other studies comparing QoL between treatment options showed comparable ranking order of the treatments to our decision model [7, 27, 28]. Which is important since our decision model assumes BCS with good cosmesis is preferred over MST (with or without reconstruction) followed by BCS with poor cosmesis. Atisha et al. and Jagsi et al. found that satisfaction with breasts as measured by Breast-Q questionnaire was highest in patients receiving MST with autologous reconstruction and slightly decreased with BCS, followed by MST with implant reconstruction, and was the lowest with MST only [7, 27]. Han et al. found that QoL and satisfaction as measured by EORTC QLQ-C30 & BR23 was higher for BCS as compared to MST or reconstructive surgery [28]. These studies suggest that current QoL differences found are clinically meaningful. It has also been found that utility increases by 0.031 if the patient is given a treatment choice versus restricting choice to MST alone [29]. Furthermore, if poor cosmesis is expected, other treatment alternatives are available, namely, neo-adjuvant chemotherapy and oncoplastic surgery including therapeutic mammoplasty. Utility studies are needed regarding these alternatives to enable implementation in a treatment decision model. This study should be interpreted as preliminary and a first step towards a more ideal treatment decision model.

The treatment decision model presented here is currently being studied in a randomized controlled trial in patients who are candidates for both BCS and MST to study the effectiveness in improving cosmetic result and QoL over the present situation [30]. Here, TV/BV ratio is measured by ultrasound instead of MRI since it is less invasive, widely available, and more cost-effective. Validation of the volume measurements is currently awaiting publication. There is a fair amount of QoL benefit (0.02 per patient) that can be expected if more research is performed that decreases parameter uncertainty as shown by our value of information analysis. Currently, we are measuring utilities in a larger study population for all health states to reduce uncertainty and improve the decision model.

## Conclusions

A common goal in breast cancer surgery including breast reconstruction is to pursue shared treatment decision making, which is currently based on informal assessment by the surgeon. We present a preliminary treatment decision model as a first step towards a more ideal treatment decision model applicable in surgical oncology. This treatment decision model can improve shared decision making in breast cancer surgery. With individual patient characteristics, namely tumor/breast volume ratio and tumor location, an expected long-term QoL value is given on a scale between 0 and 1 for both treatment options. The patient and surgeon can weigh the size of the QoL difference between BCS and MST (with or without breast reconstruction) or choose the surgery with the highest QoL. BCS results in superior QoL in patients with tumors located centrally or in the upper breast quadrants with a tumor volume/breast ratio below 15. MST with or without reconstruction is recommended for all patients with a tumor volume/breast volume ratio above 22 or tumors in the lower breast quadrants with a tumor volume/breast volume ratio above 4. Considering that the model is population-, surgeon-, and panel specific, it is advised to validate the model before introducing it in other countries.

## Electronic supplementary material

Below is the link to the electronic supplementary material.

Supplementary material 1 (DOCX 37 KB)
