# Supplementary material for: A preliminary prediction model for potentially guiding patient choices between breast conserving surgery and mastectomy in early breast cancer patients; a Dutch experience
Source: Qual Life Res. 2017 Nov 17;27(2):545–53. doi: 10.1007/s11136-017-1740-0 (PMC5846961; doi:10.1007/s11136-017-1740-0)
Supplement: Supplementary file 1 — Supplementary material 1 (DOCX 37 KB) [file 11136_2017_1740_MOESM1_ESM.docx]

A preliminary prediction model for potentially guiding patient choices between breast conserving surgery and mastectomy in early breast cancer patients; a Dutch experience. Elvira Vos, Linetta Koppert, Winnifred van Lankeren, Cornelis Verhoef, Bas Groot Koerkamp, Myriam Hunink. Department of Surgery, Erasmus MC Cancer Institute, Rotterdam, the Netherlands. Email: l.koppert@erasmusmc.nl

| Online resource 1 Erasmus MC Panel questionnaire |  |  |  |  |  |
| --- | --- | --- | --- | --- | --- |
| Overall cosmetic result |  | 0 | 1 | 2 | 3 |
| Appearance of the surgical scar | | 0 | 1 | 2 | 3 |
| Size of the breast |  | 0 | 1 | 2 | 3 |
| Shape of the breast | | 0 | 1 | 2 | 3 |
| Position of the nipple-areola complex | | 0 | 1 | 2 | 3 |
| Size of the nipple-areola complex | | 0 | 1 | 2 | 3 |
| Color of the nipple-areola complex | | 0 | 1 | 2 | 3 |
| Position of inframammary fold |  | 0 | 1 | 2 | 3 |
| Color of the skin |  | 0 | 1 | 2 | 3 |
| Teleangiectasia |  | 0 | 1 | 2 | 3 |
| Symmetry | | 0 | 1 | 2 | 3 |

0 = excellent result (without visible treatment sequelae)
1 = good result (slight sequelae with minimal difference between treated and untreated breast)
2 = fair result (obvious difference difference between treated and untreated breast but without major distortion)
3 = poor result (major aesthetic sequelae)

| Online Resource 2 Univariable and multivariable linear regression analysis for predictors of cosmetic result as evaluated by a panel^a^ | | | | |
| --- | --- | --- | --- | --- |
|  | Univariable | | Multivariable^b^ | |
|  | OR (95%CI) | P | OR (95%CI) | P |
| Age (years) | 0.01 (-0.01, 0.03) | 0.284 |  |  |
| Radiological tumor diameter (cm) | *0.20 (0.04, 0.36)* | *0.018* |  |  |
| Tumor volume/breast volume ratio (TV/BV ratio) | *0.04 (0.01, 0.07)* | *0.017* |  |  |
| Tumor location |  |  | *0.04 (0.00, 0.07)* | *0.028* |
| Upper lateral | reference |  | reference |  |
| Lower lateral | *0.51 (0.17, 0.85)* | *0.004* |  |  |
| Upper medial | -0.09 (-0.42, 0.24) | 0.587 | *0.41 (0.09, 0.74)* | *0.015* |
| Lower medial | 0.47 (-0.16, 1.09) | 0.138 |  |  |
| Central | 0.01 (-0.45, 0.47) | 0.981 | *0.61 (0.04-1.18)* | *0.036* |
| Pathological tumor diameter (cm) | 0.15 (-0.02, 0.33) | 0.090 |  |  |
| Adjacent DCIS (yes versus no) | 0.19 (-0.07, 0.46) | 0.148 |  |  |
| Specimen weight (gram) | *0.01 (0.002, 0.01)* | *0.003* | *0.004 (0.001, 0.007)* | *0.008* |
| Excision volume (g/cm3) | *0.05 (0.02, 0.09)* | *0.001* |  |  |
| Re-excision (yes versus no) | 0.27 (-0.25, 0.78) | 0.303 |  |  |
| Time of follow-up (months) | <0.01 (-0.01, 0.01) | 0.850 |  |  |

Abbreviation: TV/BV ratio=tumor volume in relation to breast volume

^a^ Cosmetic result was evaluated by the Erasmus MC Panel questionnaire (see Online resource 1). Higher score means reduced cosmetic result.

^b^ Multivariable analyses were performed by stepwise backward linear regression of all univariable predictors with P<0.05. Only the significant results are shown.

**Online Resource 3 Equations**

$Prediction model result=probability of good cosmetic result after BCS (p(BCS,good/R)=1/(1+℮^(-0.157*TV/BV ratio-2.738*(lower lateral quadrant)-1.026*(upper medial quadrant)-2.886*(lower medial quadrant)-0.262*(central location)-2.658) )$

**Equation 1**

The benefit of a true positive result as opposed to a false negative result is the benefit of identifying a patient who will have a good cosmetic result after BCS rather than undergoing mastectomy:

$Benefit=QoL(BCS,good)-QoL(mastectomy\pm reconstruction)$

**Equation 2A**

The harm of a false positive result as opposed to a true negative result is the harm caused by performing BCS with a poor cosmetic result rather than performing mastectomy: $Harm=QoL\left( mastectomy\pm reconstruction \right)-QoL\left( BCS,bad \right)$

**Equation 2B**

The odds at the treatment threshold is given by:

$odds\left( BCS,good/R \right)>harm/benefit$

Thus, the decision model suggests:

$Perform BCS if test result p(BCS,good/R)>\frac{\mathrm{Harm}}{Harm+Benefit}$

Substituting equations B.2A and B.2B in the above yields the optimal cutoff probability:

$p(BCS,good/R)>\frac{\mathrm{QoL}\left( \mathrm{mastectomy}\pm reconstruction \right)-QoL\left( BCS,poor \right)}{\mathrm{QoL}\left( BCS,good \right)-QoL\left( BCS,poor \right)}$

**Equation 3A-C**

Revising Equation 1 with the updated intercept yields:
$Prediction model result (R)=probability of good cosmetic result after BCS (p(BCS,good/R)=1/(1+℮^(-0.157*TV/BV ratio-2.738*(lower lateral quadrant)-1.026*(upper medial quadrant)-2.886*(lower medial quadrant)-0.262*(central location)-2.798) )$

**Equation 4**

$Perform BCS if: p(BCS,good/R)>\frac{0.023}{0.023+0.042}$ = 0.358

**Equation 5**

| Online Resource 4 EQ-5D utility calculations from the source QoL questionnaires (EORTC QLQ-C30 and FACT-G) from the different source populations (Vos et al., Jagsi et al., and Freedman et al.) for the current analysis | | | | | | | | | | | | | | |
| --- | --- | --- | --- | --- | --- | --- | --- | --- | --- | --- | --- | --- | --- | --- |
| Source study population | **Health state,**  **number of patients** | **Source QoL values** | | | | | | | | | | **Utilities** | | |
|  |  | EORTC QLQ C-30^a^ | | | | | | | FACT-G^b^ | | | EQ-5D before correction | EQ-5D difference | EQ-5D after correction |
|  |  | PF | EF | SF | CO | DI | PA | SL | PWB | FWB | EWB |  |  |  |
| Vos et al.^6^ | BCS Total, n=61 | 89.9 | 83.7 | 88.8 | 6.6 | 3.3 | 11.7 | 23.5 |  |  |  | 0.847^c^ |  |  |
|  | BCS Good, n=44 | 90.5 | 85.4 | 88.6 | 7.6 | 3.0 | 8.3 | 22.7 |  |  |  | 0.865^c^ | +0.018^e^ |  |
|  | BCS Poor, n=17 | 88.6 | 79.4 | 89.2 | 3.9 | 3.9 | 20.6 | 25.5 |  |  |  | 0.800^c^ | -0.047^f^ |  |
| Jagsi et al.^7^ | MST only, n=263 |  |  |  |  |  |  |  | 25.8 | 21.3 | 20.6 | 0.859^d^ |  |  |
|  | MST + reconstruction, n=222 |  |  |  |  |  |  |  | 26.9 | 22.1 | 20.8 | 0.876^d^ |  |  |
| Freedman et al.^11^ | BCS Total, n=1050 |  |  |  |  |  |  |  |  |  |  | 0.890 |  |  |
| Current analysis | BCS Good |  |  |  |  |  |  |  |  |  |  |  |  | 0.908^g^ |
|  | BCS Poor |  |  |  |  |  |  |  |  |  |  |  |  | 0.843^h^ |
|  | MST (± reconstruction) |  |  |  |  |  |  |  |  |  |  |  |  | 0.866^i^ |

Abbreviations: QoL=quality of life, BCS=breast conserving surgery, MST=mastectomy

^a^ PF: Physical Functioning, EF: Emotional Functioning, SF: Social Functioning, CO: Constipation, DI: Diarrhoea, PA: Pain, SL: Sleep

^b^ PWB: Physical Well-Being, FWB: Funtional Well-Being, EWB: Emotional Well-Being

^c^ EQ-5D utility=1-(0.8592777-0.0069693*PF-0.0087346*EF-0.0039935*SF+0.0000355*PF²+0.0000552*EF²+0.000029*SF²+0.0011453*CO+0.0039889*DI+0.0035614*PA-0.0003678*SL-0.000054*DI²+0.0000117*SL²

^d^ EQ-5D utility=0.391+(0.009*PWB)+(0.008*FWB)+(0.005*EWB) after rescaling FACT-G values on a 0-100 scale

^e^ 0.865(BCS Good)-0.847(BCS Total)= +0.018

^f^ 0.800(BCS Poor)-0.847(BCS Total)=-0.047

^g^ 0.890(BCS Total from Freedman et al.)+0.018(BCS Good EQ-5D difference from Vos et al.)=0.908

^h^ 0.890(BCS Total from Freedman et al.)-0.047(BCS Poor EQ-5D difference from Vos et al.)=0.843

^i^ reconstruction rate: 41.6%^9^ > (0.859*0.584)+(0.876*0.416)=0.866

| **Online Resource 5** Probabilistic sensitivity analysis (PSA) and expected value of perfect information (EVPI) analysis | | | | | | | | | | | | |
| --- | --- | --- | --- | --- | --- | --- | --- | --- | --- | --- | --- | --- |
| Sample | p(BCS,good) | U(BCS,good) | p(BCS,  poor) | U(BCS, poor) | **Benefit(BCS)** | p(MST only) | U(MST only) | p(MST, recon) | U(MST, recon) | **Benefit(MST)** | Treatment Advice | Opportunity Loss in Utilities |
| 1 | 0.713 | 0.913 | 0.316 | 0.772 | 0.894 | 0.540 | 0.861 | 0.441 | 0.878 | 0.852 | BCS | 0.000 |
| 2 | 0.761 | 0.914 | 0.292 | 0.862 | 0.947 | 0.599 | 0.853 | 0.397 | 0.884 | 0.862 | BCS | 0.000 |
| 3 | 0.650 | 0.917 | 0.332 | 0.851 | 0.879 | 0.596 | 0.860 | 0.417 | 0.881 | 0.880 | Mastectomy | 0.001 |
| 4 | 0.682 | 0.917 | 0.209 | 0.848 | 0.802 | 0.623 | 0.851 | 0.418 | 0.890 | 0.902 | Mastectomy | 0.100 |
| 5 | 0.719 | 0.924 | 0.244 | 0.817 | 0.863 | 0.555 | 0.841 | 0.440 | 0.876 | 0.852 | BCS | 0.000 |
| 6 | 0.621 | 0.906 | 0.324 | 0.772 | 0.813 | 0.592 | 0.867 | 0.401 | 0.878 | 0.866 | Mastectomy | 0.053 |
| 7 | 0.667 | 0.919 | 0.335 | 0.869 | 0.905 | 0.618 | 0.843 | 0.405 | 0.871 | 0.873 | BCS | 0.000 |
| 8 | 0.719 | 0.912 | 0.277 | 0.832 | 0.886 | 0.580 | 0.880 | 0.414 | 0.883 | 0.876 | BCS | 0.000 |
| 9 | 0.705 | 0.920 | 0.251 | 0.819 | 0.854 | 0.595 | 0.858 | 0.394 | 0.870 | 0.853 | BCS | 0.000 |
| 10 | 0.700 | 0.913 | 0.364 | 0.878 | 0.958 | 0.562 | 0.883 | 0.424 | 0.878 | 0.868 | BCS | 0.000 |
| Average | 0.709 | 0.908 | 0.290 | 0.844 | 0.889 | 0.584 | 0.860 | 0.416 | 0.876 | 0.866 | **61.9% BCS** | **Total EVPI** = **0.020** |
| SD | 0.055 | 0.014 | 0.055 | 0.038 | 0.069 | 0.022 | 0.013 | 0.022 | 0.015 | 0.029 |  |  |
| Abbreviations: BCS=breast conserving surgery, BCS,good=breast conserving surgery with good cosmetic result, BCS,poor= breast conserving surgery with poor cosmetic result, MST=mastectomy with or without breast reconstruction, MST only=mastectomy only, MST,recon=mastectomy with breast reconstruction.  From the total of 10,000 randomly drawn values from the beta distributions of each parameter, the first 10 simulated samples are shown in columns 2-5 and 7-10. In column 6 and 11, the net benefit in utility from respectively BCS and MST are shown. The treatment with the largest benefit is the treatment that is advised in that simulated sample (column 12). On average BCS is advised in 61.9% of the 10,000 samples. Ideally, in the presence of large amounts of data, no parameter uncertainty exists (perfect information) whereby on average 0.02 utility could be won per patient. | | | | | | | | | | | | |
